# Supplementary material for: Structural model for differential cap maturation at growing microtubule ends
Source: eLife. 2020 Mar 10;9:e50155. doi: 10.7554/eLife.50155 (PMC7064335; doi:10.7554/eLife.50155)
Supplement: Transparent reporting form [file elife-50155-transrepform.pdf]

eLIFE
1st Floor
24 Hills Road
Cambridge CB2 1JP, UK
P 01223 855340
W elifesciences.org
T @elife

eLife's transparent reporting form

We encourage authors to provide detailed information *within their submission* to facilitate the interpretation and replication of experiments. Authors can upload supporting documentation to indicate the use of appropriate reporting guidelines for health-related research (see EQUATOR Network), life science research (see the BioSharing Information Resource), or the ARRIVE guidelines for reporting work involving animal research. Where applicable, authors should refer to any relevant reporting standards documents in this form.

If you have any questions, please consult our Journal Policies and/or contact us: editorial@elifesciences.org.

Sample-size estimation

- You should state whether an appropriate sample size was computed when the study was being designed
- You should state the statistical method of sample size computation and any required assumptions
- If no explicit power analysis was used, you should describe how you decided what sample (replicate) size (number) to use

Please outline where this information can be found within the submission (e.g., sections or figure legends), or explain why this information doesn't apply to your submission:

Due to the nature of the experiments performed, no patients or animals were employed and thus, no sample size calculations were performed for the present work. Standard GLPs guidelines were followed in order to design our experiments.

Replicates

- You should report how often each experiment was performed
- You should include a definition of biological versus technical replication
- The data obtained should be provided and sufficient information should be provided to indicate the number of independent biological and/or technical replicates
- If you encountered any outliers, you should describe how these were handled
- Criteria for exclusion/inclusion of data should be clearly stated
- High-throughput sequence data should be uploaded before submission, with a private link for reviewers provided (these are available from both GEO and ArrayExpress)

Please outline where this information can be found within the submission (e.g., sections or figure legends), or explain why this information doesn't apply to your submission:

eLife Sciences Publications, Ltd is a limited liability non-profit non-stock corporation incorporated in the State of Delaware, USA, with company number 5030732, and is registered in the UK with company number FC030576 and branch number BR015634 at the address 1st Floor, 24 Hills Road, Cambridge CB2 1JP | August 2014

Biochemical data were obtained from three replicate experiments carried out in three different days using at least two samples for each condition. We made no allusion to this experimental design as they are indicated in standard GLPs.

Due to the nature of X-ray crystallography experiments, no biological replication is applicable. Structure determination was performed from a single crystal of each condition (BeF_3^- or AlF_3^-)

Fiber diffraction experiments were performed as mentioned in materials and methods. Each sample was used to obtain between 4 and 6 images and each dataset contains at least 24 independent images.

Any further information can be reported or included in the manuscript upon request.

Statistical reporting

- Statistical analysis methods should be described and justified
- Raw data should be presented in figures whenever informative to do so (typically when N per group is less than 10)
- For each experiment, you should identify the statistical tests used, exact values of N, definitions of center, methods of multiple test correction, and dispersion and precision measures (e.g., mean, median, SD, SEM, confidence intervals; and, for the major substantive results, a measure of effect size (e.g., Pearson's r, Cohen's d)
- Report exact p-values wherever possible alongside the summary statistics and 95% confidence intervals. These should be reported for all key questions and not only when the p-value is less than 0.05.

Please outline where this information can be found within the submission (e.g., sections or figure legends), or explain why this information doesn't apply to your submission:

All biochemical data are presented in **Figure 2** as Average ± Standard error.

X-ray crystallography experiments and final image statistics were calculated using the programs indicated in material and methods section. Indexed and integrated images were scaled using AIMLESS software in which completeness, redundancy, coherence (CC 1/2), single reflection significance (I/σI), data quality (R_{merge}, $R_{p.i.m.}$) and model quality indicators (R_{work}/R_{free}) are detailed in **Table 1.**

Fiber diffraction data detailed in **Table 3** are presented as Average ± Standard error. Lateral metric error calculations were performed from J_0 and J_n intensity maxima standard deviations. Longitudinal metric errors were calculated from fitting goodness to Lorentzian normal distribution function, as detailed in Material and Methods section.

Any further information can be reported or included in the manuscript upon request.

(For large datasets, or papers with a very large number of statistical tests, you may upload a single table file with tests, Ns, etc., with reference to sections in the manuscript.)

Group allocation

- Indicate how samples were allocated into experimental groups (in the case of clinical studies, please specify allocation to treatment method); if randomization was used, please also state if restricted randomization was applied
- Indicate if masking was used during group allocation, data collection and/or data analysis

Please outline where this information can be found within the submission (e.g., sections or figure legends), or explain why this information doesn't apply to your submission:

This information does not apply to our submission, as no retrospective or clinical studies were performed.

Additional data files ("source data")

- We encourage you to upload relevant additional data files, such as numerical data that are represented as a graph in a figure, or as a summary table
- Where provided, these should be in the most useful format, and they can be uploaded as "Source data" files linked to a main figure or table
- Include model definition files including the full list of parameters used
- Include code used for data analysis (e.g., R, MatLab)
- Avoid stating that data files are "available upon request"

Please indicate the figures or tables for which source data files have been provided:

